# Harnessing apomixis: natural mechanisms and synthetic innovations for advancing crop and forage breeding

**DOI:** 10.1093/hr/uhaf186

**Published:** 2025-07-22

**Authors:** Shuyi Hu, Xiaoyun Han, Lei Tian, Kejian Wang, Shuangyan Chen

**Affiliations:** State Key Laboratory of Forage Breeding-by-Design and Utilization, Key Laboratory of Plant Molecular Physiology, Institute of Botany, Chinese Academy of Sciences, Beijing 100093, China; University of Chinese Academy of Sciences, Beijing 100049, China; State Key Laboratory of Forage Breeding-by-Design and Utilization, Key Laboratory of Plant Molecular Physiology, Institute of Botany, Chinese Academy of Sciences, Beijing 100093, China; University of Chinese Academy of Sciences, Beijing 100049, China; State Key Laboratory of Forage Breeding-by-Design and Utilization, Key Laboratory of Plant Molecular Physiology, Institute of Botany, Chinese Academy of Sciences, Beijing 100093, China; University of Chinese Academy of Sciences, Beijing 100049, China; State Key Laboratory of Rice Biology and Breeding, China National Rice Research Institute, Hangzhou 310006, China; State Key Laboratory of Forage Breeding-by-Design and Utilization, Key Laboratory of Plant Molecular Physiology, Institute of Botany, Chinese Academy of Sciences, Beijing 100093, China

## Abstract

Apomixis, a reproductive mechanism that enables clonal seed production, generates progeny genetically identical to the maternal parent. In plant breeding, sexual reproduction can enhance traits through genetic recombination and hybrid vigor, yet trait segregation significantly raises breeding costs and complexity. Although apomixis occurs naturally across various plant species, it remains notably absent in major crops like rice and maize. Significant progress has been made in identifying the genes that govern this process. Recent breakthroughs in synthetic apomixis provide promising pathways for crop improvement. This review offers a comprehensive analysis of natural apomixis and its genetic regulators, with a focus on recent advances in synthetic apomictic systems. We also explore the current state and potential of apomixis in forage breeding, especially in addressing challenges related to self-incompatibility, polyploidy, and genomic complexity in forage species. Finally, we discuss the challenges in applying apomixis to forage breeding and future directions for this research.

## Introduction

In most forage species, self-incompatibility prevents self-pollination and cross-pollination with genetically identical individuals, even though the plants possess functional male and female organs [1]. This phenomenon promotes heterozygosity and genetic diversity; however, it also increases breeding costs, prolongs breeding cycles, and complicates in-depth biological studies [2–4]. Furthermore, when combined with heterosis—a phenomenon where hybrids demonstrate superior growth, resilience, and yield—retaining desirable traits across generations presents notable challenges [5, 6]. Additionally, inbreeding depression in forage species, along with trait segregation through sexual reproduction, further complicates breeding, requiring costly and labor-intensive hybridization each season [7, 8].

Apomixis has emerged as a promising alternative to address these challenges. This clonal reproductive method bypasses meiosis and fertilization to produce progeny genetically identical to the maternal plant, preserving hybrid vigor and overcoming self-incompatibility, thereby reducing the reliance on repeated hybridization. Since its discovery in *Alchornea ilicifolia*, apomixis has been documented in multiple species [9–12]; however, it remains absent in major crops such as rice (*Oryza sativa*), wheat (*Triticum aestivum*), and maize (*Zea mays*). Introducing apomixis into these staple crops could profoundly impact agriculture by enabling consistent trait inheritance through clonal seeds [8, 13, 14].

Advancements in molecular biology suggest that engineered apomixis systems are on the horizon, with the potential to revolutionize crop production and breeding practices [13–22]. This review explores recent research on apomixis, with a focus on genetic regulators and the development of synthetic apomictic systems. By synthesizing current findings, we aim to guide future research and encourage the adoption of apomixis technology in forage.

**Figure 1:**
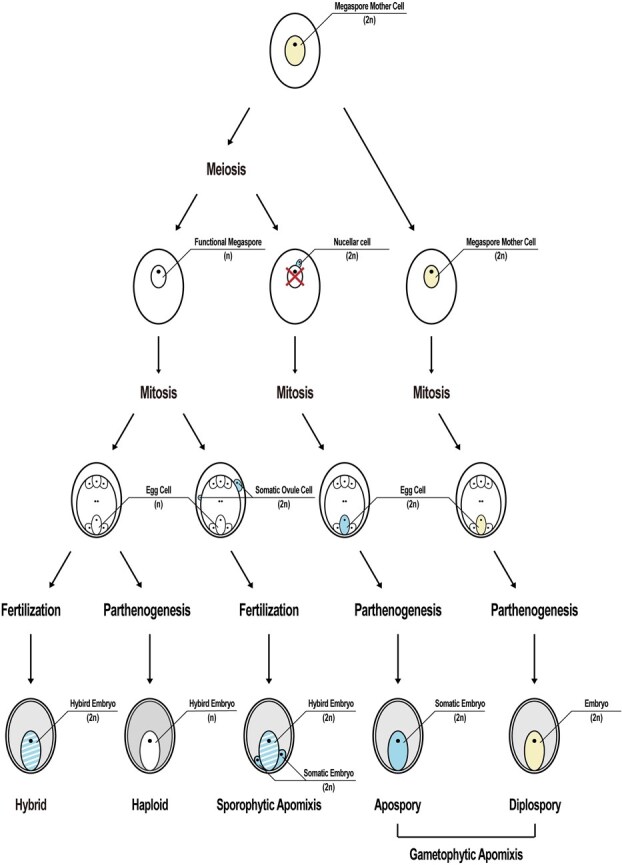
Mechanisms of sexual and apomictic seed development. This diagram illustrates the primary differences between sexual and apomictic seed development pathways, specifically highlighting sporophytic and gametophytic apomixis. Arrows indicate the developmental direction of each seed production type, while a red cross marks the replacement of a functional megaspore by somatic cells. Different colors represent cellular origins and ploidy levels: yellow for diploid cells derived from the megaspore mother cell, blue for diploid cells originating from somatic cells, blue-and-white striped for heterozygous diploid cells formed post-fertilization, white for haploid cells and tetraploid central cells, and gray for endosperm with uncertain ploidy.

## Natural apomixis

Understanding natural apomixis is essential for advancing engineered reproductive systems. Apomixis, widely observed in plants [23, 24], often coexists with sexual reproduction. Most naturally apomictic plants exhibit both sexual and asexual reproduction, reflecting a facultative reproductive strategy [25, 26]. Apomixis can be categorized into two main types based on mechanism: sporophytic apomixis and gametophytic apomixis [8, 11, 12, 25]. In sporophytic apomixis, also known as adventitious embryony, the embryo forms directly from somatic cells of the sporophyte. In contrast, gametophytic apomixis involves embryo formation from diploid somatic cells within the embryo sac. Gametophytic apomixis further divides into apospory and diplospory, defined by the origin of the embryo sac's diploid precursor cells [27]. In apospory, nucellar cells, which are somatic cells surrounding the megaspore mother cell, assume its function, whereas in diplospory, diploid precursor cells of the embryo sac develop directly from the megaspore mother cell without meiosis (Fig. 1).

**Figure 2:**
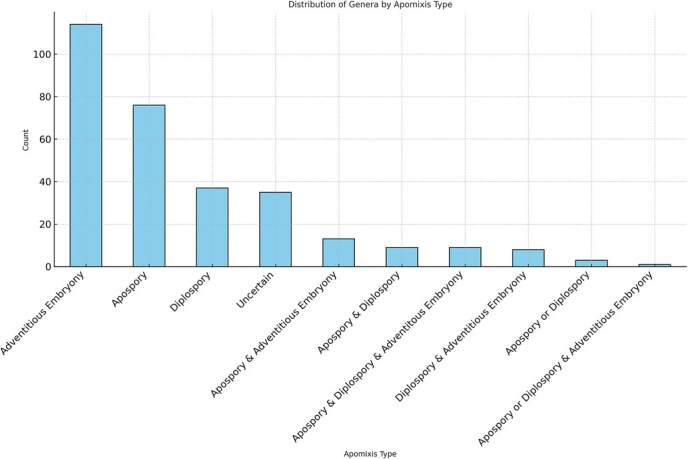
Distribution of plant genera by apomixis type. This bar graph presents the number of plant genera associated with each type of apomixis, illustrating the distribution across species. Data were sourced from the apomixis database (http://www.apomixis.unigoettingen.de).

A survey of apomictic species reveals that apomixis occurs across more than 33 orders, 77 families, 305 genera, and over 400 species of plants. However, specific data may vary due to differences in species classification and criteria for identifying apomixis [8, 14, 15, 24, 26, 28, 29]. Sporophytic apomixis has been documented in 145 genera, with 114 exhibiting only this type. Gametophytic apomixis is found in 156 genera, with 111 showing apospory and 67 displaying diplospory; of these, 76 genera exclusively exhibit apospory, while 37 exhibit diplospory. Additionally, 43 genera present multiple forms of apomixis and 35 genera remain undetermined regarding apomictic type (Fig. 2).

**Figure 3:**
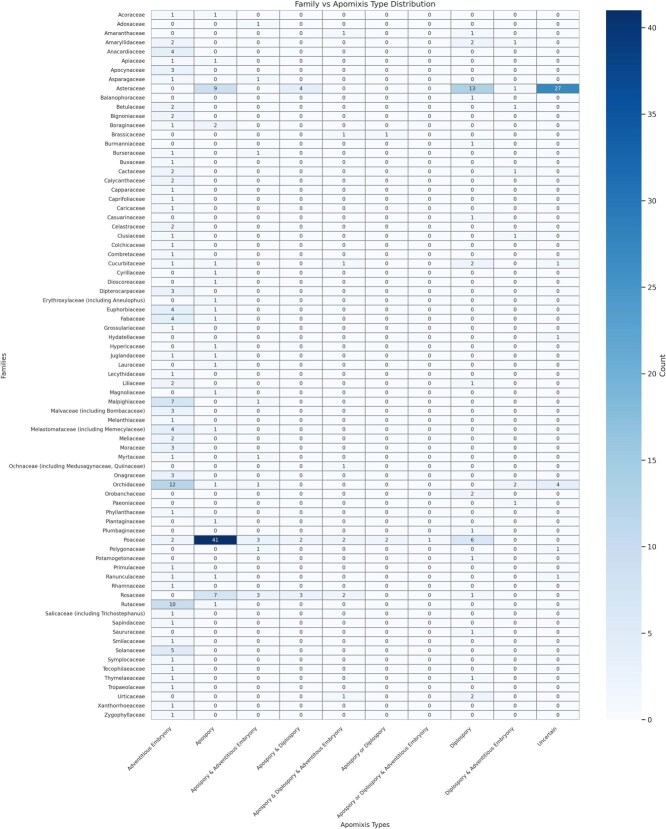
Heatmap of apomixis types among plant families. This heatmap displays the frequency distribution of apomixis types across various plant families. Darker shades indicate higher frequencies, underscoring families with stronger associations to particular apomixis types. Data were sourced from the Apomixis database (http://www.apomixis.unigoettingen.de), highlighting possible evolutionary adaptations and reproductive strategies within these families.

At the family level, Poaceae and Asteraceae are predominant, with apomictic records in 59 and 54 genera, respectively, followed by Orchidaceae (20 genera), Rosaceae (16 genera), and Rutaceae (11 genera) (Fig. 3). Notably, most families tend to prefer a single apomictic type; for example, Poaceae predominantly displays gametophytic apomixis, as does Asteraceae. However, with nearly half of Asteraceae genera unclassified by apomictic type, further investigation is necessary. Gametophytic apomixis is highly concentrated in Poaceae and Asteraceae, aligning with studies that associate polyploidy and hybridization with gametophytic modes [9, 10, 30]. Although sporophytic apomixis is more broadly distributed, it shows less association with polyploidy and hybridization, as observed in certain genera such as Citrus [31]. In traditional taxonomy, sporophytic apomixis in many species is often classified as a reproductive trait alone [30, 31].

Historically, apomixis was thought to lead to genetic uniformity within populations, reducing adaptability and resulting in an evolutionary dead end. However, this view has shifted as more apomictic species have been identified, revealing apomixis as a flexible reproductive system. Current studies indicate a global distribution of apomictic plants, often mirroring broader biodiversity patterns [32]. Apomictic plants are frequently more widely distributed than their sexually reproducing relatives, particularly in northern regions [24, 33]. Evolutionary evidence suggests apomixis has independently evolved across diverse species, with facultative apomixis providing reproductive stability over large areas and supporting mutation and selection over time [26, 28].

Due to the complexity of facultative apomixis, pathway diversity, and environmental sensitivity in apomictic plants, most studies have been limited to the genus level [9, 28]. This limitation affects data precision and hinders detailed investigation. Advancing apomixis research to the species level with innovative survey techniques, flow cytometry, advanced sequencing, and microscopy is therefore crucial for enhancing data quality and research accuracy [10]. Implementing mechanisms such as sporophytic apomixis, apospory, and diplospory in engineered contexts poses significant challenges, largely due to resource competition among concurrently developing embryos.

## Candidate genes for engineering apomixis

In sporophytic apomixis, apomictic and sexual reproduction occur simultaneously, leading to the concurrent development of adventitious and sexual embryos. This competition for limited resources at similar developmental stages can hinder the normal germination and growth of adventitious embryos, thereby limiting its potential in engineered apomixis applications [34].

Apospory, another form of apomixis, is significantly influenced by environmental and genetic factors. Somatic embryos often develop alongside sexual embryos, with the dominant developmental pathway ultimately determined by their interactions. This variability and unpredictability in aposporous development reduce its reliability for engineered applications [23]. In contrast, diplospory allows apomictic embryos to form directly from unreduced megaspore mother cells, effectively bypassing meiosis and avoiding competition between asexual and sexual embryos. This trait makes diplospory highly suitable for engineered apomixis applications [24, 30].

Given its potential, diplospory requires overcoming three main challenges: bypassing meiosis, inducing direct embryo formation from the egg cell and synchronous endosperm development [14, 35]. Specifically, meiosis must be bypassed during embryo sac formation, while egg cells should be prompted to develop independently into embryos through mechanisms such as parthenogenesis. Meanwhile, synchronous development of the endosperm with the apomictic embryo is essential for successful apomixis. Identifying the genes that enable these processes is crucial for advancing apomixis technology in major crops. Although advances in haploid breeding have identified many relevant genes, numerous genes with high potential for haploid induction remain underexplored in apomixis research. Additionally, research on autonomous endosperm development is limited, its mechanism is unclear, and gene—regulation efforts have had minimal success [15]. Thus, it is not discussed further in this paper (Table 1).

**Table 1:** Genes with potential applications in apomictic reproduction and their associated functions.

Component of apomixis	Gene	Source species	Functional verification species	Gene product/function	Specific functions in apomixis	References
Apomeiosis						
	*BVF1*	*O. sativa*	*O. sativa*	Bivalent formation 1	Lack of recombination	([36], [37])
	*CRC1*	*O. sativa*	*O. sativa*	Central region component 1	Lack of recombination	[38]
	*DYAD/SWI1*	*A. thaliana*	*A. thaliana*	DYAD/Switch1	Single-gene-induced clonal diploid gamete production	([39], [40])
	*DFO*	*A. thaliana*	*A. thaliana*	DSB formation	Lack of recombination	[41]
	*MTOPVIB*	*A. thaliana*	*A. thaliana*	Meiotic topoisomerase VI B-like	Lack of recombination	[42]
	*MTOPVIB*	*Hordeum vulgare*	*H. vulgare*	Meiotic topoisomerase VI B-like	Lack of recombination	[43]
	*MTOPVIB*	*Z. mays*	*Z. mays*	Meiotic topoisomerase VI B-like	Lack of recombination	[44]
	*MTOPVIB*	*O. sativa*	*O. sativa*	Meiotic topoisomerase VI B-like	Lack of recombination	[45]
	*PAIR1*	*O. sativa*	*O. sativa*	Homologous pairing aberration in rice meiosis1	Lack of recombination	([46], [20], [19], [17], [21], [18], [47])
	*PRD1*	*A. thaliana*	*A. thaliana*	Putative recombination initiation defect 1	Lack of recombination	[48]
	*PRD1*	*O. sativa*	*O. sativa*	Putative recombination initiation defect 1	Lack of recombination	[49]
	*PRD1*	*Z. mays*	*Z. mays*	Putative recombination initiation defect 1	Lack of recombination	[50]
	*PRD2*	*A. thaliana*	*A. thaliana*	Putative recombination initiation defect 2	Lack of recombination	([51], [48])
	*PRD2*	*O. sativa*	*O. sativa*	Putative recombination initiation defect 2	Lack of recombination	[52]
	*PRD3*	*A. thaliana*	*A. thaliana*	Putative recombination initiation defect 3	Lack of recombination	([51], [48])
	*PRD3*	*Z. mays*	*Z. mays*	Putative recombination initiation defect 3	Lack of recombination	[53]
	*SDS*	*O. sativa*	*O. sativa*	Synaptonemal complex defective in sporulation 1	Lack of recombination	[54]
	*SPO11–1*	*A. thaliana*	*A. thaliana*	Sporulation11–1	Lack of recombination	([55], [56], [48], [57])
	*SPO11–1*	*O. sativa*	*O. sativa*	Sporulation11–1	Lack of recombination	([48], [57], [22])
	*SPO11–1*	*Solanum lycopersicum*	*S. lycopersicum*	Sporulation11–1	Lack of recombination	[58]
	*SPO11–2*	*A. thaliana*	*A. thaliana*	Sporulation11–2	Lack of recombination	[59]
	*SPO11–2*	*O. sativa*	*O. sativa*	Sporulation11–2	Lack of recombination	[60]
	*SPO11–2*	*Z. mays*	*Z. mays*	Sporulation11–2	Lack of recombination	[61]
	*RDR6*	*O. sativa*	*O. sativa*	RNA-dependent RNA polymerase 6	Lack of recombination	[62]
	*REC8*	*A. thaliana*	*A. thaliana*	Meiotic recombination protein 8	Loss of co-segregation of sister chromatids	([56], [48], [57], [63], [40])
	*REC8*	*O. sativa*	*O. sativa*	Meiotic recombination protein 8	Loss of co-segregation of sister chromatids	([64], [20], [17], [19], [21], [18], [47], [22])
	*REC8*	*S. lycopersicum*	*S. lycopersicum*	Meiotic recombination protein 8	Loss of co-segregation of sister chromatids	[58]
	*OSD1*	*A. thaliana*	*A. thaliana*	Omission of second division	Skipping of the second meiotic division	([56], [48], [63], [40])
	*OSD1*	*O. sativa*	*O. sativa*	Omission of second division	Skipping of the second meiotic division	([48], [20], [17], [19], [21], [65], [47], [22])

(Continued)

**Table 1:** Continued

Component of apomixis	Gene	Source species	Functional verification species	Gene product/function	Specific functions in apomixis	References
	*TAM/CYCA1;2*	*A. thaliana*	*A. thaliana*	Tardy asynchronous meiosis/cyclin A1;A2	Skipping of the second meiotic division	([57], [63])
	*TAM/CYCA1;2*	*S. lycopersicum*	*S. lycopersicum*	Tardy asynchronous meiosis/cyclin A1;A2	Skipping of the second meiotic division	[58]
	*TDM1*	*A. thaliana*	*A. thaliana*	Three division mutant 1	Skipping of the second meiotic division (AtTDM1-P17L mutation)	([63], [66])
*Abnormal Fertilization*						
	*CENH3*	*A. thaliana*	*A. thaliana*	Centromeric histone H3	Paternal gene elimination in haploid induction and apomixis	([40], [67])
	*CENH3*	*Triticum aestivum*	*T. aestivum*	Centromeric histone H3	Paternal gene elimination in haploid induction	[68]
	*CENH3*	*Z. mays*	*Z. mays*	Centromeric histone H3	Paternal gene elimination in haploid induction	[69]
	*MTL/PLA1/NLD*	*O. sativa*	*O. sativa*	Matrilineal/phospholipase A1/Not Like DAD	Paternal gene elimination in haploid induction and apomixis	([20], [70], [22])
	*PLA1/NLD/MTL*	*Z. mays*	*Z. mays*	Phospholipase A1/not like DAD/matrilineal	Paternal gene elimination in haploid induction and apomixis	([71], [72], [73])
	*PLD3*	*Z. mays*	*Z. mays*	Phospholipase D3	Paternal gene elimination in haploid induction	[74]
	*POD65*	*Z. mays*	*Z. mays*	Peroxidase 65	Paternal gene elimination in haploid induction	[75]
	*pPLAIIγ*	*A. thaliana*	*A. thaliana*	Gynoecium-expressed phospholipase AII	Expressed in maternal tissue influencing fertilization	[76]
	*GEX1*	*Z. mays*	*Z. mays*	Gamete expressed protein 1	Expressed in maternal tissue influencing fertilization	[77]
*Parthenogenesis*						
	*ECS*	*A. thaliana*	*A. thaliana*	Egg cell-specific aspartic endopeptidase	Induced parthenogenesis in haploid induction	([78], [79])
	*ECS*	*O. sativa*	*O. sativa*	Egg cell-specific aspartic endopeptidase	Induced parthenogenesis in haploid induction	[79]
	*ASGR-BBML*	*Pennisetum squamulatum*	*P. squamulatum, P. glaucum, O. sativa, Z. mays*	Apospory-specific genomic region—baby boom like1	Induced parthenogenesis in haploid induction	([80], [81], [82])
	*BBM1*	*O. sativa*	*O. sativa*	Baby boom1	Induced parthenogenesis in haploid induction and apomixis	([19], [17], [65])
	*BBM1*	*Z. mays*	*Z. mays*	Baby boom1	Induced parthenogenesis in haploid induction and apomixis	[83]
	*BBM2*	*O. sativa*	*O. sativa*	Baby boom2	Induced parthenogenesis in haploid induction and apomixis	([17], [21])
	*BBM3*	*O. sativa*	*O. sativa*	Baby boom3	Induced parthenogenesis in haploid induction and apomixis	([17], [21])
	*BBM4*	*O. sativa*	*O. sativa*	Baby boom4	Induced parthenogenesis in haploid induction and apomixis	([17], [21])
	*PAR*	*Taraxacum officinale*	*T. officinale, Lactuca sativa, O. sativa*	Parthenogenesis	Induced parthenogenesis in haploid induction and apomixis	([84], [16], [18])
	*WUS/MOC3*	*O. sativa*	*O. sativa*	Wuschel/Monoculm 3	Induced parthenogenesis in apomixis	[47]

In research on meiosis bypass, key genes like *DYAD/SWITCH1* (*SWI1*), which encodes a protein essential for chromatid cohesion and chromosome structure during meiosis, have been identified. In *Arabidopsis thaliana*, mutations in *DYAD* lead to triploid seeds, produced through fertilization of a haploid male gamete with an unreduced female gamete, illustrating how a single-gene mutation can effectively induce meiosis bypass. However, this mutation is 99.9% sterile on the female side [39]. Similarly, the homologous gene *AMEIOTIC1* (*AM1*) in maize and rice exhibits comparable sterility issues [85, 86]. In addition, in Boechera species with natural apomixis, the 5′UTR region of the *Apomixis-Linked Locus* (*APOLLO*) gene exhibits apomixis-specific polymorphisms. The *APOLLO* gene in apomictic individuals contains a 20-bp insertion in the 5′UTR region, which is absent in the sexual form of the gene. This insertion causes the *APOLLO* gene to be highly expressed only in the developing egg cells of apomictic individuals and not in sexual individuals. However, when transformed into *Arabidopsis*, the gene may be influenced by a different transcription factor landscape, leading to its expression in somatic and not reproductive tissues [87].

While such genes can independently bypass meiosis, each has its own issues. Moreover, the combined action of multiple genes—those affecting recombination, sister chromatid co-segregation, and the second meiotic division—is thought to promote the conversion of meiosis to mitosis, generating clonal diploid gametes, and is considered a viable approach [15, 88].

Firstly, *Homologous Pairing Aberration in Rice Meiosis1* (*PAIR1*) in rice controls homologous chromosome pairing during the first meiotic division, with mutations resulting in abnormal chromosome segregation [46]. The homologous gene *Putative Recombination Initiation Defect* (*PRD*) plays a similar role in other species [49–53]. The Sporulation11 (SPO11) protein, which catalyzes DNA double-strand breaks (DSB) in *Saccharomyces cerevisiae*, is conserved in plants and initiates meiotic recombination, highlighting its importance across species [55, 59–61, 89]. The *Meiotic Topoisomerase VI B-like* (*MTOPVIB*) gene has also been implicated in DSB formation during meiosis and forms a complex with SPO11, collectively promoting the initiation of meiotic recombination [42–45]. In addition to the aforementioned genes, *DSB Formation* (*DFO*), *Central Region Component 1* (*CRC1*), *Synaptonemal Complex Defective in Sporulation 1* (*SDS*), *Bivalent Formation 1* (*BVF1*), and *RNA-dependent RNA Polymerase 6* (*RDR6*) are also implicated in DSB formation and can influence recombination [36–38, 41, 54, 62].

Secondly, *Meiotic Recombination Protein 8* (*REC8*) and *Sister Chromatid Cohesion 3* (*SCC3*), crucial for centromere cohesion. These proteins operate in meiosis independently of SPO11. However, *SCC3* is not only essential in meiosis but also crucial in mitosis. Mutations in *SCC3* lead to chromosome behavior abnormalities during mitosis, which in turn cause abnormal vegetative growth in plants [64, 90].

Thirdly, *Omission of Second Division* (*OSD1*) inhibits anaphase promoting complex/cyclosome (APC/C) to regulate meiosis. In Arabidopsis and rice, knocking out *OSD1* bypasses the second meiotic division, yielding diploid gametes [17–20, 40, 47, 48, 56, 65]. Similarly, mutations in *Tardy Asynchronous Meiosis* (*TAM*) and *Three Division Mutant 1* (*TDM1*), which regulate meiotic exit, also induce these phenotypes [57, 58, 63, 66]. However, it should be noted that knockouts of the *TDM1* gene only result in a third meiotic division, whereas only specific point mutations, such as *AtTDM1-P17L*, can bypass the second cell division [63, 91].

After obtaining clonal diploid gametes by influencing the meiosis process, clonal diploid seeds can primarily be induced through abnormal fertilization and parthenogenesis [15, 23, 32, 34].

Abnormal fertilization pathways primarily involve combinations of clonal diploid gametes and genes affecting fertilization to induce haploid formation [15, 20, 34, 40]. For instance, *Centromeric Histone H3* (*CENH3)* can induce gradual paternal genome loss during post-fertilization embryogenesis, a highly conserved mechanism in plants that may also cause chromosome elongation in approximately 30% of cases [67–69, 92, 93]. The *Phospholipase A1*/*Matrilineal*/*Not Like DAD* (*PLA1*/*MTL*/*NLD*) gene, which encodes a sperm-specific phospholipase localized to the peri-germ cell membrane from a vegetative cell, can also induce paternal genome elimination [20, 70–73, 94]. Additionally, while not yet confirmed, *Phospholipase D3* (*PLD3*) and *Peroxidase 65* (*POD65*) may contribute to sperm DNA fragmentation and thus hold potential for applications in this context [74, 75]. Unlike the previously mentioned genes, other genes, such as *gynoecium-expressed phospholipase AII* (*pPLAIIγ*) and *Gamete Expressed protein 1* (*GEX1*), which are expressed in the maternal tissues, can induce haploid formation by influencing fertilization pathways [76, 77].

While abnormal fertilization pathways offer one method to induce plant formation from clonal diploid gametes, another strategy involves bypassing fertilization entirely through parthenogenesis. This mechanism leverages specific genes to directly trigger embryo development in egg cells, circumventing the need for fertilization. Within the apospory-specific genomic region (ASGR) of *Pennisetum squamulatum*, the *ASGR–Baby Boom-Like* (*ASGR-BBML*) gene has been identified as capable of bypassing the fertilization checkpoint in egg cells to induce apomixis. This gene shares high homology with the *BABY BOOM* (*BBM*) gene, which is known as an APETALA 2 (AP2)/ETHYLENE RESPONSIVE FACTOR (ERF) domain transcription factor. When ectopically expressed in monocot egg cells, *PsASGR-BBML* has successfully induced haploid formation [81–83, 95]. However, in dicots, the ability of *PsASGR-BBML* to induce parthenogenesis is limited; ectopic expression in Arabidopsis failed to produce haploid progeny [80, 81]. Further research into parthenogenesis repressors in Arabidopsis identified that mutations in the transcription factor *RWP-RK Domain Protein 5* (*RKD5*) enhance *AtBBM* expression and can induce a limited number of haploids [96]. Another natural parthenogenesis-related gene *Parthenogenesis* (*PAR*), isolated from apomictic dandelion (*Taraxacum officinale*), has successfully induced parthenogenesis through ectopic and heterologous expression in rice, lettuce (*Lactuca sativa*), and foxtail millet (*Setaria italica*) [16, 18, 84, 97]. Moreover, the genes *Egg Cell-Specific Aspartic Endopeptidase* (*ECS*), which encode egg cell-specific endopeptidases, have demonstrated the ability to induce maternal haploid production via a partial double fertilization process called pseudogamy [78, 79]. In addition, recent studies have revealed that the *Wuschel* (*WUS*) gene in rice, which regulates tiller bud formation and development and is associated with somatic embryogenesis and embryo development, can also induce parthenogenesis when ectopically expressed in egg cells [47, 98, 99]. This discovery is significant as previous research on the *WUS* gene had not linked it to the induction of parthenogenesis, highlighting the diversity and potential of genes that can induce parthenogenesis.

In addition, some genes can boost the induction efficiency of haploid genes by acting as inducers. For instance, co-expressing *WUSCHEL-Related Homeobox 9A* (*WOX9A*) and *BBM1* in rice egg cells can achieve a parthenogenesis rate of 86%–91%, which is 5–16 times higher than that from ectopic expression of *BBM1* alone [100]. Similarly, in maize with *MTL* mutations, the *DOMAIN OF UNKNOWN FUNCTION 679 membrane protein* (*DMP*) mutation can increase the haploid induction rate by 5–6 times [101].

Identifying and validating key genes linked to apomixis is fundamental for advancing engineered apomixis technology, with expanding the gene pool being essential for further advancements. While substantial progress has been made in identifying and applying apomixis-related genes, many gene functions remain insufficiently understood, which limits their full application potential. Relying solely on the existing gene pool is unlikely to overcome all technical barriers. Thus, apomixis technology development relies heavily on haploid breeding techniques and research on meiotic mechanisms. Drawing insights from haploid breeding and exploring methods to utilize haploid-inducing genes for apomixis applications represent effective approaches to broadening the gene pool for apomixis research and applications.

## Engineering apomixis

Despite significant progress in identifying and validating numerous apomixis-related genes, only a limited number have been successfully implemented in engineered systems due to substantial practical challenges.

To address the sterility resulting from single-gene mutations that bypass meiosis, key breakthroughs have been achieved in Arabidopsis by creating a triple mutant with mutations in *SPO11–1*, *REC8*, and *OSD1*, effectively substituting meiosis with a mitosis-like process [56]. Notably, this substitution did not compromise later stages of sexual reproduction. Building on this success, the team developed the ‘Mitosis instead of Meiosis’ (*MiMe*) approach, which presents a promising pathway for bypassing meiosis through genetic engineering. Subsequent studies demonstrated that the creation of a *TDM1*-*REC8*-*OSD1* triple mutant also enabled the *MiMe* pathway [63, 66]. These findings suggest that substituting key genes in *MiMe* mutants with functional analogs is a viable strategy.

**Figure 4:**
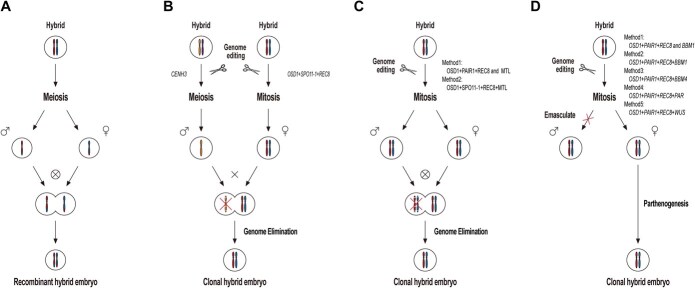
Comparison of sexual reproduction and engineered apomixis pathways. (A) Natural sexual reproduction. (B) Mitosis instead of Meiosis (*MiMe*) crossing with the *CENH3* tailswap line (*GEM*). (C) *MiMe* combined with abnormal fertilization to induce apomixis. (D) *MiMe* combined with parthenogenesis to induce apomixis. Arrows depict the developmental direction for each pathway, with red crosses marking chromosome elimination in the zygote development process or the blockage of specific pathways. The “⊗” symbol indicates self-pollination and The “×” symbol denotes hybridization. The “+” symbol connecting genes indicates that they are constructed on the same vector, whereas “and” between genes denotes separation on different vectors. For instance, “*OSD1+PAIR1+REC8* and *MTL*” implies that *OSD1*, *PAIR1*, and *REC8* are on one vector, while *MTL* is on a separate vector. These vectors are transformed independently to produce mutants.

Although *MiMe* mutants have successfully generated clonal diploid male and female gametes, progeny resulting from natural reproduction are frequently tetraploid, with an increasing ploidy trend over generations. Therefore, further refinement of engineered apomixis technology requires a mechanism to directly induce gametophyte development into new individuals. Building on this, researchers used *MiMe* triple mutants of *Arabidopsis* as maternal parents, crossing them with the *CENH3* tailswap line (*GEM*). Unlike natural sexual reproduction (Fig. 4A), this method results in progeny with complete maternal genetic identity [40] (Fig. 4B). This accomplishment marked the first experimental realization of engineered apomixis technology. However, it is important to note that artificial hybridization is still required to produce clonal progeny, making this a semi-autonomous genome elimination apomixis system. Although *CENH3* has been used to successfully induce haploids in many plants, only in *Arabidopsis* has it been reported to induce apomixis. But as *CENH3* can cause chromosome elongation, this method of inducing apomixis is not ideal [22, 67–69].

Following the success of the *MiMe* approach in *Arabidopsis*, researchers have begun efforts to extend this technology to crops, particularly rice. As previously noted, detailed studies of the *MiMe* system in *Arabidopsis* have demonstrated the feasibility of constructing new *MiMe* triple mutants by substituting the original genes with functional analogs. Developing an *MiMe* system in rice shows promise; however, despite the high homology of *SPO11–1*, *REC8*, and *OSD1* genes in rice, directly using these homologs in constructing an *MiMe* system was not preferred, likely due to unique characteristics of the rice homolog *OsSPO11–1*. Nonetheless, researchers successfully established an *MiMe* system in rice using *OsSPO11–1*, *REC8*, and *OSD1* [22]. With further research into the *PAIR1*, *REC8*, and *OSD1* genes in rice, the first rice *MiMe* system was constructed through the combination of mutations in these threegenes, achieving fertility comparable to the control group [48]. While the *MiMe* systems have been successfully created in rice and *Arabidopsis*, their extension to other plants has faced challenges. This is mainly because in *Arabidopsis* and rice, *OSD1* is redundant with its homolog *Ultraviolet-B-Insensitive 4* (*UVI4*). In contrast, plants like tomato (*Solanum lycopersicum*) possess a single *UVI4* copy, which is implicated in mitosis. This makes it challenging to obtain a homozygous *OSD1* mutant suitable for the *MiMe* process [58]. However, researchers established an *MiMe* system in tomato by creating a triple mutant of *SPO11–1*, *REC8*, and *TAM* recently. This system produces diploid pollen (36.75%–62.18%) and results in tetraploid tomato plants [58]. But efforts to create apomictic plants in cotton (*Gossypium hirsutum*) by manipulating *REC8*, *PAIR1*, *OSD1* genes and ectopically expressing the BBM gene have been unsuccessful. Despite the production of transgenic plants, their fertility was significantly reduced [102]. In soybean (*Glycine max*), CRISPR-Cas9-mediated knockout of meiotic genes (*GmSPO11–1*, *GmREC8A/B*, and *GmOSD1A/B*) was used to induce an *MiMe* system. While these genes showed conserved functionality with their *Arabidopsis* and rice orthologs, the mutants generated aneutetraploids with chromosome loss rather than the expected tetraploids. The observed small-scale chromosomal abnormalities indicate that diploid clonal gamete production might still be achievable through targeted suppression of meiotic errors [103].

In rice haploid breeding research, mutations in the *MTL* gene induced maternal haploid induction rates of 2%–6% [70]. Using CRISPR/Cas9, researchers introduced mutations in *MTL* and the *MiMe* genes into hybrid rice, resulting in quadruple mutants (*OsPAIR1*, *REC8*, *OSD1*, and *MTL*), termed the ‘Fix’ mutant [20]. During self-pollination, these mutants successfully induced clonal progeny with stable clonal traits over generations. However, fertility was low at 4.5%, and clonal induction rates remained between 6%–8% [20]. This strategy was also validated in rice the *MiMe* systems constructed using *SPO11–1*, *REC8*, and *OSD1* genes, though issues with low seed setting and clonal induction rates persisted [22]. Unlike the *CENH3*-mediated apomixis method in *Arabidopsis*, this approach autonomously induces paternal genome elimination through self-pollination, creating an engineered apomictic system. Nevertheless, due to the characteristics of *MTL* and other genes involved in abnormal fertilization, this system is limited to low-frequency clonal seed production, thereby restricting its further development [15].

Concurrently, researchers successfully induced clonal progeny with parthenogenetic characteristics by ectopically expressing the *BBM1* gene in the egg cells of *MiMe* mutants, achieving an induction rate of 10%–30% [17] (Fig. 4C). This method, which directly generates clonal progeny from egg cells, shows better fertility and higher clonal progeny induction rates compared to the *MTL*-induced method. However, the seed setting and induction rates still fall short of practical requirements. To enhance this technology, researchers further combined *PAIR1*, *REC8*, *OSD1*, and *BBM1* genes on a single T-DNA vector, achieving one-step engineered production of F1 hybrid seeds using CRISPR/Cas9 technology (Fig. 4D). This approach maintained 30% fertility and achieved a high clonal seed frequency (>80%), with the trait remaining stable across multiple generations [19]. Subsequently, utilizing a similar one-step hybrid seed construction method, researchers combined the *BBM1* homolog *BBM4*, *BBM3*, and *BBM2* with *PAIR1*, *REC8*, and *OSD1*, respectively, to generate three types of quadruple mutants. Among them, the *BBM4* and *BBM2* lines achieved normal fertility (>80%), despite a lower clonal progeny induction rate (<3%) [21]. In subsequent modifications, based on the one-step hybrid seed construction method, the SunTag gene activation system was employed to enhance the expression of *OsBBM1* driven by the *ECA1.1* promoter in rice. This approach achieved clonal rates exceeding 95% in some lines, with fertility comparable to wild-type plants [65] (Fig. 4D). These findings represent a significant advance in engineered apomixis and have encouraged further exploration of alternative parthenogenetic pathways.

In addition, researchers have developed an apomictic reproduction system in rice, induced by the endogenous *WUS* gene, which maintained normal fertility while achieving a seed clonality rate of 21.7% [47] (Fig. 4D). Inspired by these advances, scientists have recently enhanced the apomixis system in rice by introducing genes from naturally apomictic plants. For example, introducing the *PAR* gene from dandelion into rice successfully induced apomictic lines, not only achieving clonal seed induction rates between 42.9%–67.7% but also maintain normal fertility [18] (Fig. 4D).

In summary, current engineered apomixis approaches can be broadly categorized into three types: [104] a semi-autonomously induced paternal genome elimination system represented by *GEM* lines, [105] an autonomously induced paternal genome elimination system represented by the *MTL* line, and [95] an autonomously induced parthenogenesis system represented by the *BBM* line. Although these methods have significantly advanced engineered apomixis, the conflict between fertility and clonality rate remains a primary obstacle to practical application. Building on previous developments, future breakthroughs in engineered apomixis may involve incorporating genes from naturally apomictic plants, screening for novel candidate genes from established haploid induction gene libraries, and innovatively combining these genes through effective methodologies.

### The current status of apomixis in forage crops

Natural apomixis occurs in some forage grasses, like species in the genera *Poa*, *Brachiaria*, and *Paspalum*, with many diplospory cases reported. Yet, the genes controlling apomixis are still poorly understood [10, 29, 30]. Research so far has focused on roughly mapping the apomixis-specific genomic region using molecular markers and speculatively identifying gene functions [106–109]. However, in recent years, there has been a rise in efforts to introduce apomixis into other forage and vegetable crops, such as alfalfa (*Medicago sativa*), sorghum (*Sorghum bicolor*), and cowpea (*Vigna unguiculata*), with several teams working in this area [110].

In our recent investigation of *Leymus chinensis* seed germination, a significant frequency of polyembryony was observed in the analyzed samples (unpublished data). This phenomenon is strongly associated with apomixis and has been proposed as a detectable phenotype for screening apomictic mutants [35, 111]. Although apomixis has not been previously documented in *L. chinensis* or the genus *Leymus*, our findings suggest its potential presence in this species.

**Figure 5:**
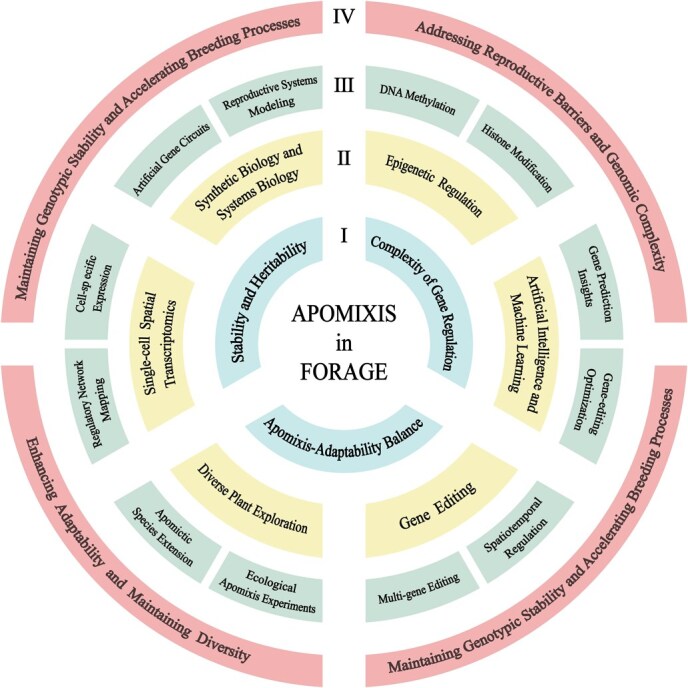
Future challenges, breakthroughs, and potential applications of apomixis in forage crops. I challenges in advancing apomixis research; II emerging technologies contributing to apomixis research; III potential breakthroughs on the horizon; IV applications of apomixis in forage crop breeding.

This observation raises the possibility that apomixis may be more widespread in forage crops than currently documented. Several lines of evidence support this hypothesis. First, gametophytic apomixis is strongly associated with allopolyploidy [9, 10], a genetic characteristic prevalent in many forage species. Second, forage crops are predominantly members of the Poaceae family, which has been consistently identified across multiple studies as having a high incidence of apomixis [8, 15, 24, 26, 28, 29]. Third, despite its potential significance, research on apomixis in forage crops remains limited [106–109]. Taken together, these factors suggest that apomixis may be more common in forage crops than currently recognized, with numerous cases likely remaining undocumented.

Apomixis holds immense potential for agricultural crop production, particularly in forage crops. This reproductive mechanism enables seed production without genetic recombination or trait segregation, thereby overcoming challenges such as self-incompatibility and inbreeding depression [112]. By facilitating the rapid fixation of desirable traits, reducing dependence on manual or cross-pollination, shortening breeding cycles, and lowering seed production costs, apomixis has the potential to revolutionize forage breeding programs [8]. A notable example is the *BBM* gene, a key player in apomixis induction, which was first identified in the forage grass *Pennisetum squamulatum* [81]. This discovery highlights the value of forage grasses as a genetic resource for apomixis research and suggests that further exploration of apomixis-related genes in these species could expand the pool of candidate genes for apomixis induction in crops.

Despite its potential, progress in apomixis research for forage grasses has been hindered by limited basic research on growth and developmental processes, as well as low gene-editing efficiency. While successful apomixis induction has been achieved in model species such as *Arabidopsis*, rice, and tomatoes through the testing of various gene combinations, similar efforts in forage grasses remain challenging. However, recent advancements in gene-editing technologies, coupled with the success of the *MiMe* systems in tomatoes and the achievement of high clonal rates and fertility in rice, suggest that apomixis induction in forage grasses may soon become a reality [58, 65, 113].

## Conclusion and prospects

Apomixis, a naturally occurring reproductive mechanism in various plant species, represents a highly intricate process regulated by complex genetic pathways, epigenetic modifications, environmental cues, and molecular interactions. The artificial induction and stabilization of apomixis hold transformative potential for agriculture by enabling the production of genetically uniform, high-yield crops. However, significant technical obstacles persist, particularly in achieving precise genetic control and expanding the range of applicable plants. Despite significant progress, the fundamental mechanisms governing apomixis remain only partially understood [8]. It is essential to address these critical knowledge gaps to unlock its full potential and achieve apomixis in forage crops (Fig. 5).

A primary challenge in engineering apomixis in forage species stems from their inherent genetic complexity. The regulation of apomixis involves multiple interdependent genes and sophisticated signaling pathways, necessitating the development of comprehensive and integrative regulatory models to ensure the stability and heritability of apomictic traits across generations. However, the transfer of regulatory models from model species such as rice and *Arabidopsis* to other plants, including tomato, is hindered by significant genetic and developmental differences. These challenges are further exacerbated in forage grasses, which exhibit greater genetic complexity and diverse reproductive mechanisms compared to model species. Additionally, while apomixis offers substantial improvements in breeding efficiency, its potential to reduce genomic diversity raises concerns about the long-term adaptability and resilience of forage crops to changing environmental conditions [110]. This is particularly critical given the distinct growing conditions of forage crops compared to traditional crops, necessitating a careful balance between breeding efficiency and genetic diversity preservation.

Addressing the challenges of apomixis in forage crops requires a multifaceted approach that integrates comparative genomics, functional studies, and the development of species-specific regulatory models. Such strategies are crucial for harnessing the benefits of apomixis while mitigating its potential risks in crop improvement. For example, CRISPR-based genome editing has successfully targeted apomixis-related genes in rice and *Arabidopsis* [17–22, 40, 47, 48, 56, 57, 63, 64], demonstrating the potential of precise genetic interventions. Additionally, the integration of *BBM* and the *MiMe* genes into a single vector has significantly enhanced cloning efficiency and seed set, highlighting the importance of vector design and co-transformation strategies [17, 19, 21]. Despite these advances, further improvements in multi-gene editing and regulatory network optimization are essential to boost induction efficiency and stabilize apomictic traits over multiple generations [114].

Parallel advancements in high-resolution technologies, such as single-cell omics and spatial transcriptomics, are providing unprecedented insights into the cellular and molecular processes underlying apomixis [104, 105, 115]. These tools facilitate the identification of key regulatory elements and molecular mechanisms, while epigenetic regulation—including DNA methylation, histone modifications, and RNA silencing—offers additional layers of control over the initiation and maintenance of apomixis [116–118]. Future studies should prioritize understanding the dynamic changes in these epigenetic marks during apomictic development and exploring chemical and genetic interventions to modulate them effectively.

To further enhance research efforts in apomixis for forage crops, the integration of cutting-edge methodologies is critical. Synthetic biology provides unprecedented opportunities to design artificial regulatory circuits [119], which can be complemented by systems biology approaches that model and predict complex biological networks to identify critical pathways involved in apomixis [120]. Artificial intelligence and machine learning add another dimension by enabling the analysis of large-scale gene networks, predicting novel regulatory factors, and refining gene-editing strategies, thereby accelerating research progress [121]. Together, these approaches offer a synergistic framework for addressing the multifaceted challenges of apomixis in forage.

Notably, much of the current research on apomixis has focused on a limited number of model plants, such as *Arabidopsis* and rice, as well as select major crops [15, 48, 56, 58, 65]. Expanding investigations to include a broader range of plant species and ecological contexts will be crucial for understanding the environmental factors influencing apomixis and developing innovative strategies for both agricultural improvement and biodiversity conservation [23]. By bridging these knowledge gaps and leveraging emerging technologies, future research can unlock the full potential of apomixis, transforming agriculture and advancing our understanding of plant reproductive biology.

## Data Availability

Data sharing is not applicable to this article as no new data were created or analyzed in this study.
